# Bacterial Bile Metabolising Gene Abundance in Crohn's, Ulcerative Colitis and Type 2 Diabetes Metagenomes

**DOI:** 10.1371/journal.pone.0115175

**Published:** 2014-12-17

**Authors:** Alain Labbé, Jorge G. Ganopolsky, Christopher J. Martoni, Satya Prakash, Mitchell L. Jones

**Affiliations:** 1 Biomedical Technology and Cell Therapy Research Laboratory, Department of Biomedical Engineering, Faculty of Medicine, Montreal, Quebec, Canada; 2 Micropharma Limited, Montreal, Quebec, Canada; Cincinnati Children's Hospital Medical Center, University of Cincinnati College of Medicine, United States of America

## Abstract

We performed an analysis to determine the importance of bile acid modification genes in the gut microbiome of inflammatory bowel disease and type 2 diabetic patients. We used publicly available metagenomic datasets from the Human Microbiome Project and the MetaHIT consortium, and determined the abundance of bile salt hydrolase gene (*bsh*), 7 alpha-dehydroxylase gene (*adh*) and 7-alpha hydroxysteroid dehydrogenase gene (*hsdh*) in fecal bacteria in diseased populations of Crohn's disease (CD), Ulcerative Colitis (UC) and Type 2 diabetes mellitus (T2DM). Phylum level abundance analysis showed a significant reduction in Firmicute-derived *bsh* in UC and T2DM patients but not in CD patients, relative to healthy controls. Reduction of *adh* and *hsdh* genes was also seen in UC and T2DM patients, while an increase was observed in the CD population as compared to healthy controls. A further analysis of the *bsh* genes showed significant differences in the correlations of certain Firmicutes families with disease or healthy populations. From this observation we proceeded to analyse BSH protein sequences and identified BSH proteins clusters representing the most abundant strains in our analysis of Firmicute *bsh* genes. The abundance of the *bsh* genes corresponding to one of these protein clusters was significantly reduced in all disease states relative to healthy controls. This cluster includes *bsh* genes derived from *Lachospiraceae, Clostridiaceae, Erysipelotrichaceae* and *Ruminococcaceae* families. This metagenomic analysis provides evidence of the importance of bile acid modifying enzymes in health and disease. It further highlights the importance of identifying gene and protein clusters, as the same gene may be associated with health or disease, depending on the strains expressing the enzyme, and differences in the enzymes themselves.

## Introduction

The gut microbiome is important for multiple metabolic functions, including the absorption and production of nutrients and the development of the immune system. Variations in the bacterial populations due to differences in diet, host genetics and pathological states are currently being characterized for the development of diagnostics and therapeutics. Some of the diversity in the microbiota is however potentially eclipsed by changes in the overall gut microbial gene pools, found to be more stable in individuals, but where changes are possibly more relevant to host health. Metagenomics is the study of the total microbial genomic DNA isolated from an environmental sample, and when applied to human fecal samples provides a catalogue of all microbial genes present in the gut. Examining changes to this profile of genes is likely to yield meaningful contrast between health and disease but also provide mechanistic information on the role of certain microbial genes in host metabolism [1]–[4].

Bile acids (BAs) are amphipathic molecules that serve as detergents to facilitate the absorption of fatty molecules into circulation. The interaction of bile acids (BAs) and the intestinal microbiota within the gastrointestinal (GI) tract is important for the physiology of the host. Deconjugation of bile acid (BA) conjugates by the bacterial bile salt hydrolase (BSH) enzyme is the first step in the generation of secondary BAs. Subsequently, the action of bacterial dehydroxylase (ADH) and dehydrogenase (HSDH) activities on deconjugates in the GI leads to the production of BAs with altered capacity to enter cells, transmit signals and re-enter the enterohepatic circulation [5], [6].

Bile acid signalling is mediated by two main receptors: an intracellular nuclear receptor, Farnesoid X Receptor (FXR) and a G-protein coupled receptor (TGR-5). FXR signalling is especially important in the control of bile acid synthesis through induction of fibroblast growth factor 19 (FGF-19) in the intestine and inhibition of the activity of cholesterol 7 alpha-hydroxylase (CYP7A1). TGR-5 signalling has been associated with a reduction of immune responses through inhibition of macrophage function and in glucose regulation through increased secretion of GLP-1, directly impacting insulin secretion and post-prandial regulation of glycemia [7]–[10].

Changes in BAs and BA metabolism have been linked to both inflammatory bowel disease (IBD) (Crohn's disease (CD) and ulcerative colitis (UC)) as well as diabetes[7], [11]–[13]. Changes in the microbiome populations have also been implicated in IBD [14]–[17]. Among potential mechanisms, the changes in microbiota may lead to altered BA transformation in the gut and changes in circulating BA. Direct enzyme activity assays in IBD patients showed impaired deconjugation, dehydroxylation and desulfation [12], [18]. Observational studies have further shown that BAs are important in Type 2 diabetes [19]. BAs directly affect glucose homeostasis by repressing hepatic gluconeogenesis and enhancing glycogen synthesis, both mechanisms reducing post-prandial glycemia. In addition to effects in the liver, BA signals through TGR-5 are thought to affect metabolism by increasing cAMP in brown adipose tissue, and through stimulation of incretins, GLP-1 and GIP from L-cells in the gut [7], [19]–[21].

In the present study, we made use of large available datasets containing metagenomic information from populations of patients with IBD, diabetes and controls individuals to determine whether there are differences in the abundance of bacterial genes involved in the deconjugation (*bsh*), dehydroxylation and epimerization (*adh* and *hsdh*) of bile acids within the diseased microbiomes. We associated these changes in gene abundances with specific bacterial phylum and families, and further correlated the diseases with subgroups of genes based on clustering of BSH protein sequences.

## Material and Methods

### Datasets

Datasets were obtained from NCBI SRA database under accessions #ERP000108 (MetaHIT), SRP002427, SRP015779, SRP002423, SRP000319 and SRP011011. Average sequencing depth is presented in S1 Table. When required, the data was selected to meet the length requirements for our searches, set at 75bp to include most of the MetaHIT dataset. Metadata was also extracted from the SRA database or from publications depending on availability. S1 Table presents the description of the datasets used in our analysis. The SRA files obtained were used directly by the scripts and converted to fasta files using NCBI SRA toolkit version 2.2.2b, specifically fastq-dump with –fasta option.

### Sequence database

Genbank protein search for “bile salt hydrolase” yielded 6627 sequences and these sequences were used for our primary and secondary blast searches. The same search was performed for 7alpha-dehydroxylase and the search yielded 2900 sequences identified as 7alpha-hydroxysteroid dehydrogenase (HSDH) and 7-dehydroxylase (ADH). Only 3 sequences were identified by the 7-dehydroxylase search and these sequences were further searched independently from the main HSDH database. The sequences accessions are provided in S3 Table and include both *BaiA1* and *BaiA2* genes. *BaiA1* is a monocistronic gene while *BaiA2* is part of the polycistronic *bai* operon[6]. Protein reference database (refseq_protein) was obtained and used locally for searches.

### Search procedures

All DNA sequences from SRA files were searched, using ncbi-blast-2.2.27 blastx, against a database including both BSH and HSDH protein sequences at 35% identity and 24 aa length, representing the maximum length of the translated Illumina sequences from the MetaHIT dataset. The same conditions were used for all the datasets to allow for comparisons without introducing a bias in the BLAST search based on search length. The list of hits was used to extract the original sequence again from the SRA files and the sequences were then further searched against individual protein databases, again using blastx, for BSH, HSDH or ADH at increasing stringency. The hit sequences were saved in intermediate files and the original illumina sequence of the hits was extracted from the SRA files and searched, using blastn, against the all.db database from NCBI, a database containing non-redundant bacterial genomes found on NCBI. Hits above 50% identity and 75 bp in length were kept. All BLAST searches used default settings. The blastn hits ID and HSP sequences were saved and the IDs were searched against the NCBI taxonomy database. We confirmed the validity of the hit assignment by using tblastx, to align the translated hits sequences against a translated database of the bacterial species sequenced from the human microbiome project. We confirmed that more than 99% of hits obtained by blastn are similarly allocated by tblastx. Taxonomy information was extracted, saved to file, and counted to obtain frequency information on the origin of the *bsh*, *hsdh* or *adh* genes at the taxonomic phylum and family levels. A confirmation of the hits was done by extracting the sequences matching the all.db sequences with flanking regions of 100 bp upstream and downstream from the 75bp Illumina sequence, and searching, using blastx, for hits in the refseq_protein database from NCBI. All steps of the analysis were scripted using PERL programming language and run on Ubuntu 12.04LTS plateforms.

### Clustering

BSH protein sequences were obtained from NCBI and clustered using Clustalw 2.1 installed locally. Tree visualization was done using FigTree version1.3.1 (Institute of Evolutionary Biology, University of Edinburgh).

### Statistics

Statistical analysis was performed using SPSS ver 10 and R (ver 3.0.2). Non normally distributed data was analysed using Mann Whitney U for pairwise comparisons and Kruskall wallis for multiple comparisons. Spearman Correlation coefficient was calculated to establish relationship between bacterial family abundance of the *bsh*, *hsdh* or *adh* genes with disease. Plotting of the graphs was done using R, Excel and SPSS.

## Results

We have performed an extensive analysis of the available datasets on NCBI for UC, CD, and a dataset of Chinese diabetic patients. We used a sequential BLAST methodology to extract homologous sequences from short reads and quantified the number of hits to the bile salt hydrolase genes from a library including all annotated bile salt hydrolases from NCBI Protein database. We used progressively stricter criteria and completed the analysis using 75% identity over a 24 amino acid length. The amino acid fragment length was selected as the maximum length that could be obtained from the 75 bp sequencing length of the illumina reads for the MetaHIT consortium data. We used 75% identity to detect proteins highly similar to BSH and which may represent yet uncharacterized and annotated *bsh* genes. The DNA sequences from the hits were further searched against the all.db database of references genomes from NCBI to obtain taxonomic information. Although we used our own scripts, this approach is based on widely used methods using reference genomes for taxonomic assignments. Further, as we are first selecting hits corresponding to a specific protein (BSH, HSDH or ADH), our analysis is similar to that used by platforms such as CloVR-Metagenomics[22], providing functional assignment by aligning to a protein database and taxonomy information by matching the open reading frames to a bacterial genome database. The abundance analysis was performed on the taxonomic phyla and families, to reduce the likelihood of misassignment at the species level. The number of hits was converted to abundance in hits per million of sequences searched in the experiments, to normalize for the various samples sequencing depths. We also performed an analysis of the hit flanking regions in the reference genomes and searched the resulting database against the NCBI Refseq protein database to quantify the specificity of our analysis. We obtained an average of 80.7% of sequences correctly identified as bile salt hydrolase, an average of 6.7% hypothetical proteins and 12.6% of other sequences.

### Bsh, adh and hsdh gene abundance in IBD gut microbiome

We performed an analysis between the abundance of the *bsh* gene from various bacterial families with inflammatory bowel disease. As some of the datasets are relatively small, we combined the results from multiple available datasets on UC and CD and included a dataset of a lean-obese twin study as further non IBD controls for the analysis.

**Figure 1:**
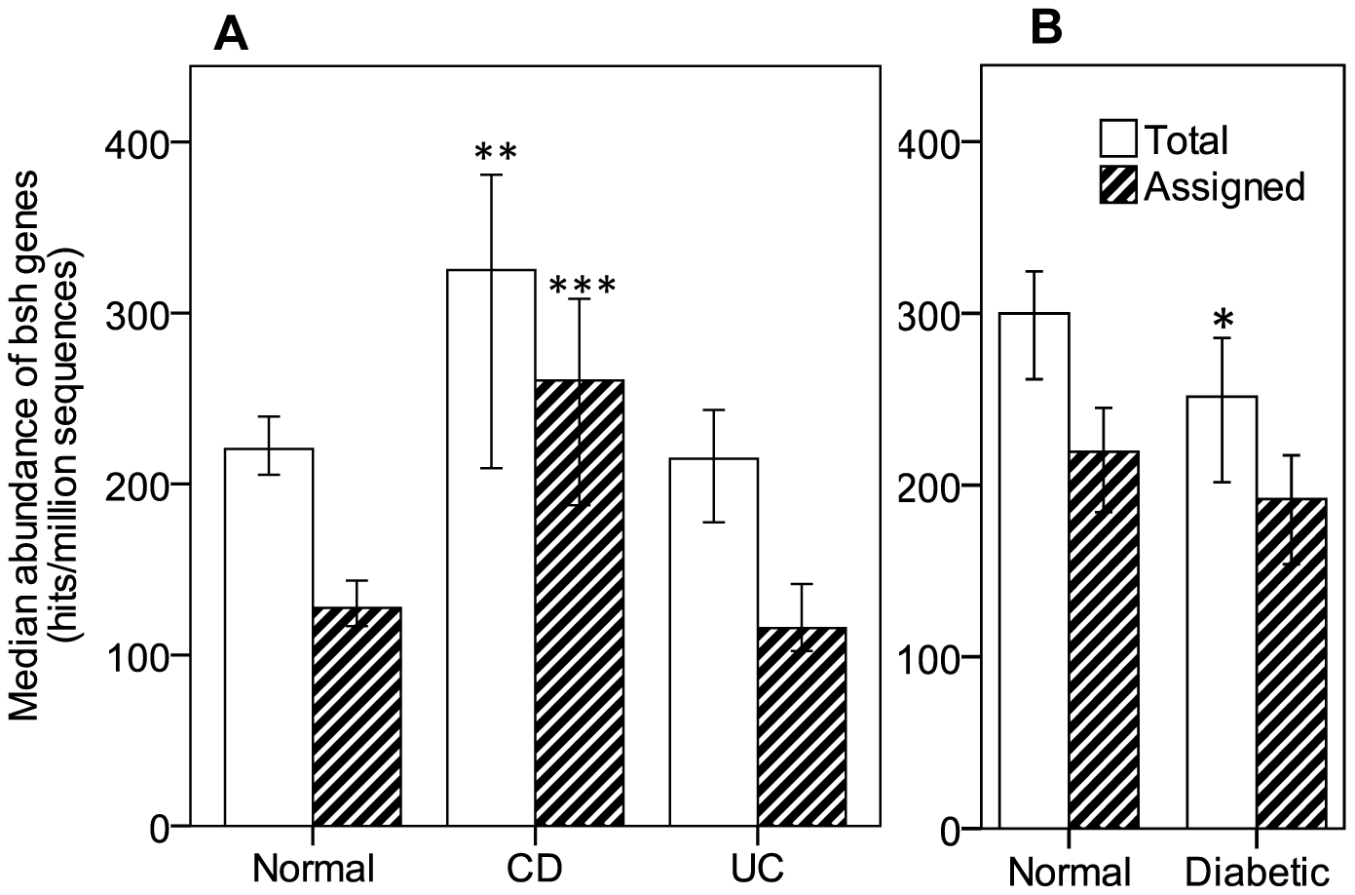
Abundance of *bsh* in metagenomic data from IBD and diabetic patients. Abundance of *bsh* genes was obtained by searching a BSH protein database against publicly available SRA sequences from HMP and MetaHIT metagenomic datasets for Crohn's Disease (CD) and Ulcerative Colitis (UC) (A) and diabetic patients (B). Hits (≥24 amino acids and ≥75% ID) were counted (Total hits) and further searched against a bacterial genome database to assign taxonomic origins to the hits (Assigned hits). Assigned and total hits were quantified and expressed as hits per millions sequences for each individual patient. Values are median abundance and error bars are 95% confidence interval. Statistical analysis was Kruskall-wallis (A) or Mann-Whitney U (B). *p<0.05, **p<0.01, ***p<0.001.

Our analysis was performed on many available datasets of metagenome data from NCBI, including “A human gut microbial gene catalog established by deep metagenomic sequencing”, METAHIT[3], “Dysfunction of the intestinal Microbiome in Inflammatory Bowel Disease and Treatment”, (SRP015779_CD)[16], “The Role of the Gut Microbiota in Ulcerative Colitis, whole metagenome sequencing project”, (SRP002427_UC), “Metagenomic Analysis of the Structure and Function of the Human Gut Microbiota in Crohn's Disease”, (SRP002423_CD)[23], and “A core gut microbiome in obese and lean twins”, (SRP000319)[24]. We combined the datasets to provide a more realistic evaluation of the specific *bsh* gene origins in CD and UC, reducing the likelihood that the observed variability be caused by specific patient populations or geographic origins. We did observe significant differences in the mean abundance of *bsh* genes in the normal, UC and CD groups, as well as a difference in the number of hits that could be assigned to specific taxonomic groups. Fig. 1A presents the median values, both of total abundance and taxonomically identified hits. We observed a significant increase in median number of hits in CD, with no significant changes between normal and UC patients (Kruskall-Wallis, normal: *n* = 114, mean rank (MR)  =  76.84, CD: *n* =  21, MR  =  110.48, UC: *n* = 25, MR  =  72.00, Normal vs CD: *p*  =  0.007). A significant increase can also be seen in the taxonomically assigned hits between normal and CD (*p*  =  0.000) patients, but again, no significant changes between the normal and UC populations. When we separately analyse the data from the HMP projects and the MetaHIT data (with patients originating from Denmark and Spain), we only detect a significant change in the abundance based on taxonomically assigned hits (*p*  =  0.005) in the HMP populations with no significant differences detected in the MetaHIT dataset between normal, CD and UC groups (Kruskal-Wallis, *p*  =  0.698 for Total hits and *p*  =  0.056 for assigned hits abundance). Individual dataset results are presented in S2 Table. “Median Abundance of *bsh* in metagenomic data from individual datasets”. We observe that the trend is found in most datasets but that significance is difficult to achieve with the limited number of samples available.

**Figure 2:**
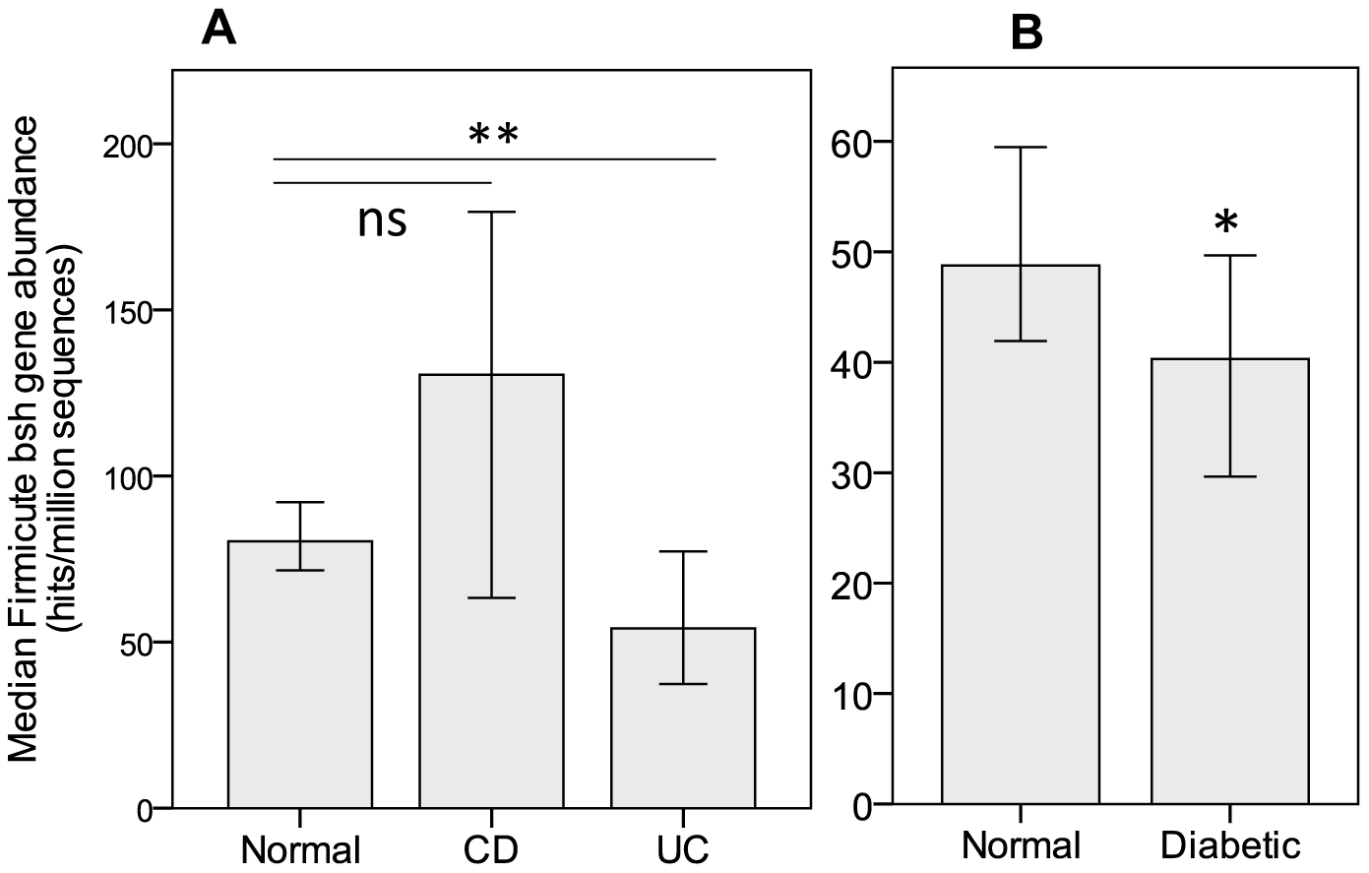
Abundance of Firmicute derived *bsh* in metagenomic data. Abundance of *bsh* genes was obtained by searching a BSH protein database against publicly available SRA sequences from HMP and MetaHIT metagenomic datasets for IBD and diabetic patients. Hits (≥24 amino acids and ≥75% ID) were counted and searched against a bacterial genome database to assign taxonomic origins to the hits. Hits assigned to the Firmicute phylum were counted and expressed as hits per millions sequences for each individual patient. Values are median abundance and error bars are 95% confidence interval. Statistical analysis was Kruskall-wallis (A) or Mann-Whitney U (B). *p<0.05, **p<0.01.

From the data obtained with our analysis, we specifically quantified the hits derived from the main bacterial phyla: Firmicutes, Bacteroidetes, Actinobacteria and Proteobacteria. A Kruskall-Wallis analysis of the abundance of the *bsh* genes in IBD indicated significant differences between normal and disease states within the Firmicute, Actinobacteria and Proteobacteria phyla while no differences could be observed in Bacteroidetes. The quantification of Firmicute derived *bsh* genes (Fig. 2A) shows a significant reduction in UC patients (Kruskall-Wallis, *p* = 0.006, normal: *n*  =  114, mean rank (MR) 83.15, UC: *n*  =  25, MR 51.40) with a non-significant increase in CD patients (*p* = 0.328). *Bsh* genes derived from Actinobacteria and Proteobacteria were increased in CD patients (Kruskall-Wallis, *p* = 0.000 and *p* = 0.005) but not in UC patients (data not shown).

**Figure 3:**
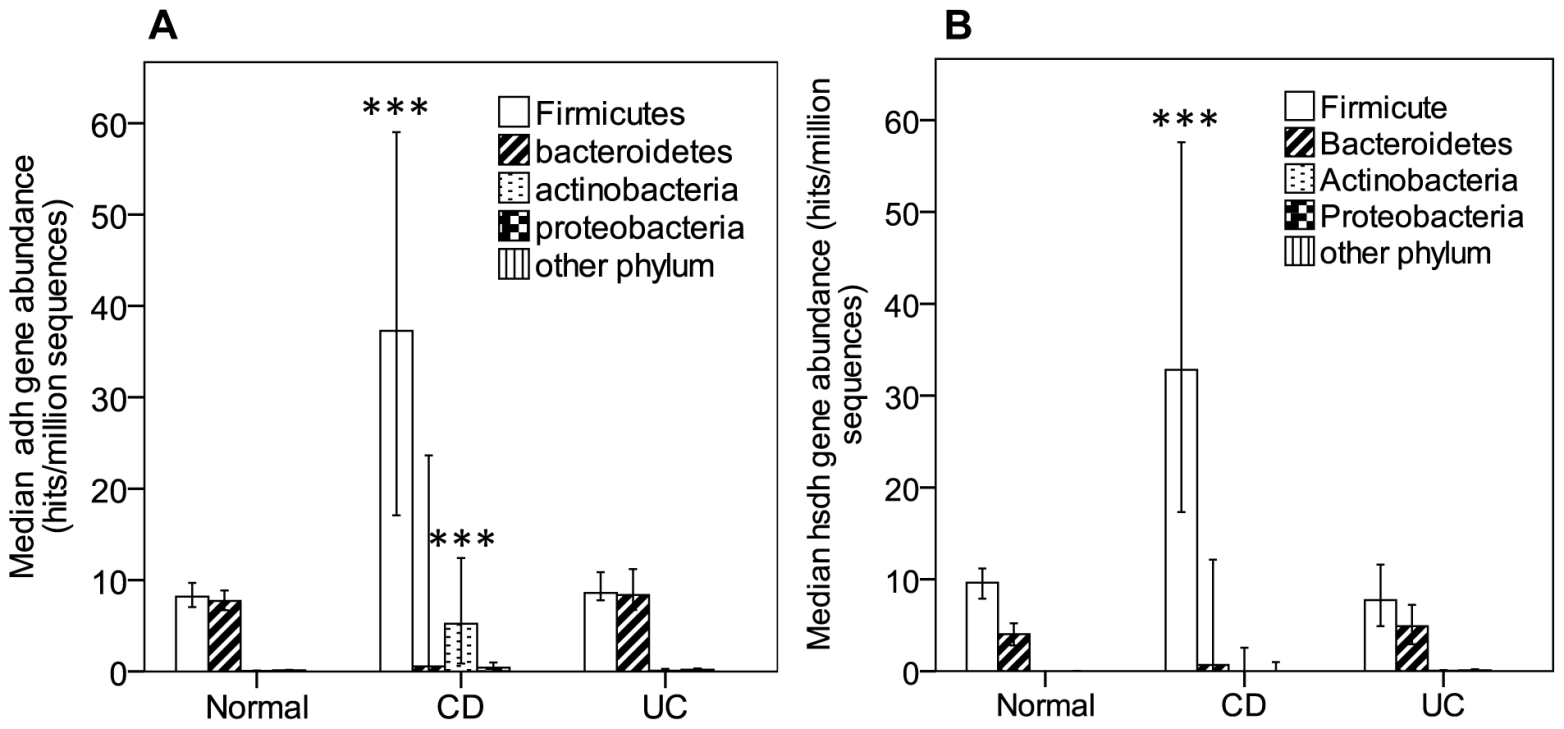
Abundance of *adh* and *hsdh* in bacterial phyla. Abundance of *adh* genes (please refer to S3 Table for sequences assigned as *adh*) (A) and *hsdh* genes (B) was obtained by searching protein database against publicly available SRA sequences from HMP and MetaHIT metagenomic datasets for IBD (UC and CD) and diabetic patients. Hits (≥24 amino acids and ≥75% ID for *hsdh* and ≥60% ID for *adh*) were counted and searched against a bacterial genome database to assign taxonomic origins to the hits. Hits assigned to the various phylum were counted and expressed as hits per millions sequences for each individual patient. Values are median abundance and error bars are 95% confidence interval. Statistical analysis was Kruskall-wallis. ***p<0.001

In addition to the evaluation of *bsh* gene abundance, we analysed for the presence of genes annotated as 7-alpha dehydroxylase (*adh*) (S3 Table) and 7-alpha hydroxysteroid dehydrogenase (*hsdh*), important enzymes in the production of secondary BAs (deoxycholic acid (DCA) and lithocholic acid (LCA)) and in the epimerization of bile acids (including one step in transition from chenodeoxycholic acid (CDCA) to ursodeoxycholic acid (UDCA)), respectively. In an analysis of our IBD dataset, we observed a significant increase of the overall abundance in taxonomically assigned *adh* genes in CD patients as compared to controls (normal: MR  =  69.39, CD: MR  =  144.10, UC: MR  =  77.76, normal vs CD: *p*  =  0.000, normal vs UC: *p*  =  1) (data not shown). The same tendencies were observed in comparisons of normal subjects with CD patients and UC patients in the abundances of Firmicute-derived *adh* (Kruskall-Wallis, normal: *n*  =  114, mean rank (MR) 72.49, UC: *n*  =  25, MR 74.72, CD: *n*  =  21, MR 130.86, normal vs CD: *p*  =  0.000, normal vs UC: *p*  =  1) and in the actinobacteria-derived *adh* (normal: MR  = 74.19, CD: MR  =  126.48, UC: MR  =  70.64, normal vs CD: *p*  =  0.000, normal vs UC: *p*  =  1) while finding no increases in UC patients (Fig. 3a). It is interesting to note that we are observing the presence of these genes (*adh*) in Bacteroidetes despite the fact that the activity has not been observed in this phylum[6]. Searches for the sequences from our *adh* database (S3 Table) does however yield matches in all 4 phylums: Firmicutes, Bacteroidetes, Actinobacteria and Proteobacteria. The significance of this finding remains to be determined. The analysis for *hsdh* yielded similar observations as observed with *adh*. It is important to note that due to the homology found between the 3alpha, 7alpha and 12alpha hydroxysteroid dehydrogenase, it is difficult to confirm whether the changes are related only to 7alpha-hydroxysteroid dehydrogenase despite the fact that our protein sequence database was selected for that gene. We found a significant increase in Firmicute-derived *hsdh* genes in CD patients relative to controls (Kruskall-Wallis, normal: *n*  =  114, mean rank (MR) 76.79, UC: *n*  =  25, MR 64.16, CD: *n*  =  21, MR 124.10, normal vs CD: *p*  =  0.000, normal vs UC: *p*  =  1) but no other significant changes in Bacteroidetes, Actinobacteria and Proteobacteria (Fig. 3b).

### Bsh, adh and hsdh gene abundance in diabetic gut microbiome

An analysis of the Chinese diabetic dataset[25] for *bsh* gene abundance shows a significant difference in the total number of hits between normals and diabetics, though no differences in the number of assigned hits was observed (Fig. 1B). (Mann-Whitney U, *p*  =  0.02 for Total hits, *p*  =  0.113 for assigned hits). As performed for UC and CD datasets, we quantified the hits derived from the main bacterial phyla: Firmicutes, Bacteroidetes, Actinobacteria and Proteobacteria. The analysis shows a reduction of Firmicute-derived BSH as compared to the control group (Mann-Whitney U, *p*  =  0.020, normal: *n*  =  100, MR  =  109.43, diabetic: *n*  =  99, MR  =  90.47) (Fig. 2B). Analysis of *bsh* gene derived from Bacteroidetes, Actinobacteria and Proteobacteria didn't show significant changes between normal subjects and diabetic patients. Analysis for *adh* and hsdh yielded significant reductions of both Firmicute-derived genes in the diabetic population (Mann-Whitney U, *p*  =  0.006, normal: *n*  =  100, MR  =  111.12, diabetic: *n*  =  99, MR  =  88.77 and *p*  =  0.026, normal: *n*  =  100, MR = 103.20, diabetic: *n*  =  99, MR  =  96.77 respectively)(data not shown).

### Firmicute families Bsh gene abundance in UC, CD and diabetic gut microbiome

**Figure 4:**
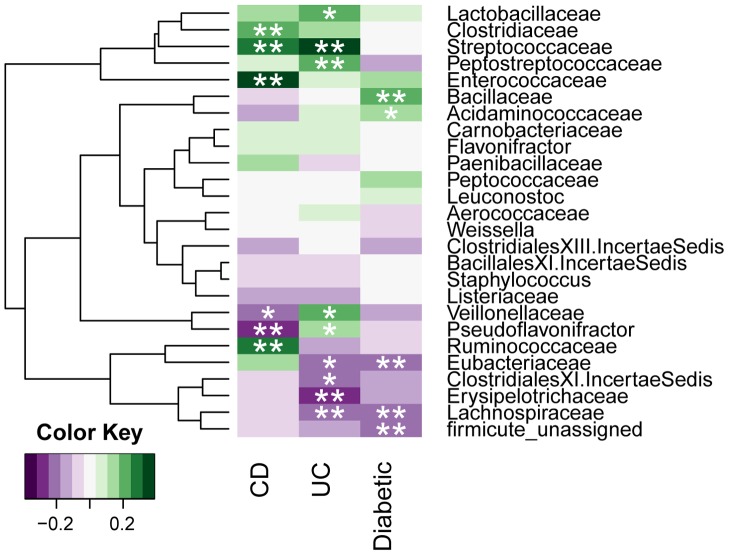
Correlation between *bsh* abundance from bacterial families and disease. Spearman's rank order correlations was performed and represented using a heatmap to determine the association of bacterial families associated *bsh* gene abundance with disease in CD, UC and diabetes. Firmicute_unassigned represents hits for which only phylum assignment was available from NCBI taxonomy database. *p<0.05, **p<0.01.

We performed a Spearman's Rank-order correlation analysis of the Firmicutes families associated with *bsh* genes and observed both positive and negative correlations with disease (Fig. 4, *p<0.05, **p<0.01). A small cluster of 4 families is especially interesting as it correlates negatively with disease in UC, CD and diabetes.

**Figure 5:**
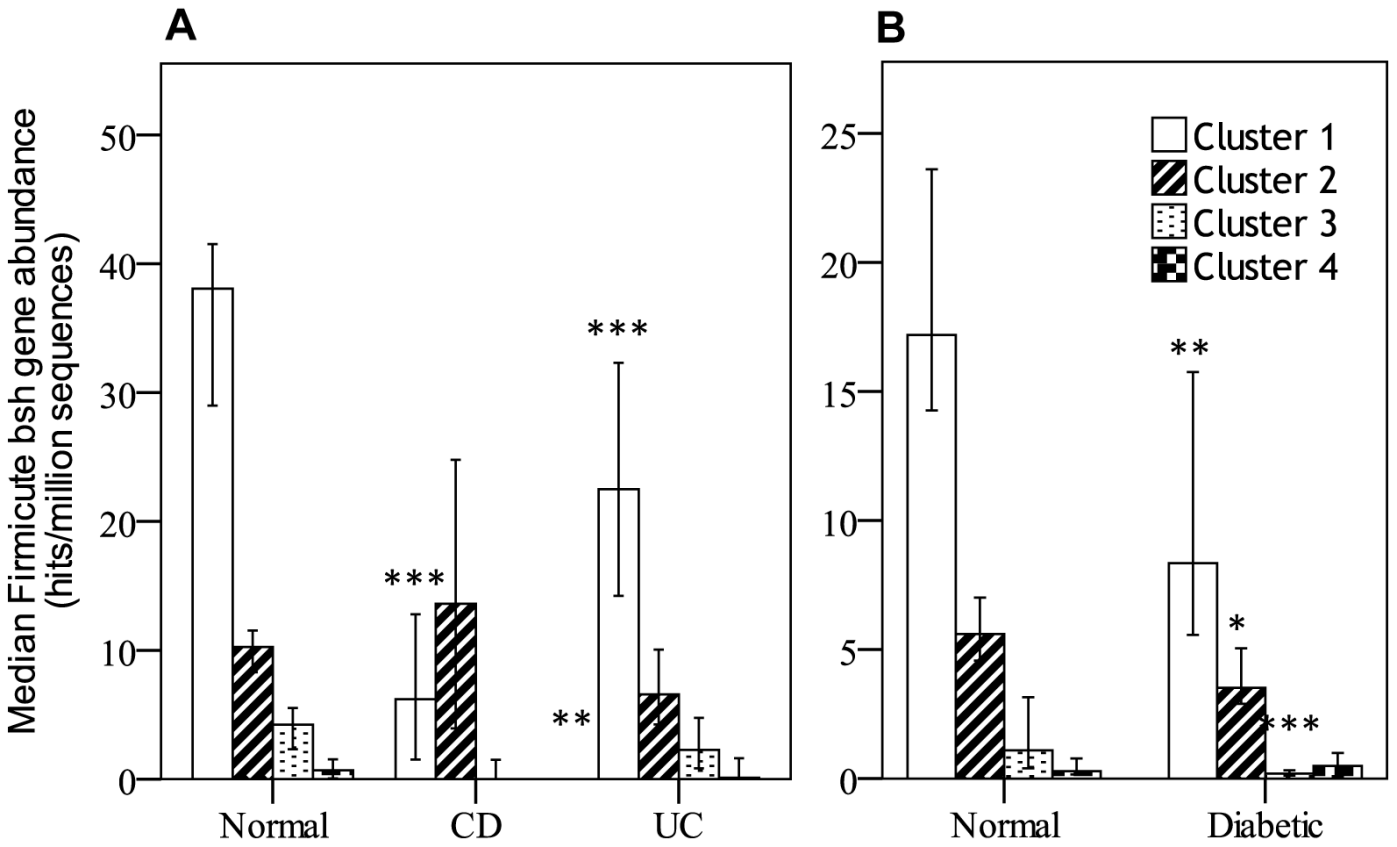
Abundance of Firmicute derived *bsh* by BSH protein clusters. Abundance of *bsh* genes was obtained by searching a bsh protein database against publically available SRA sequences from HMP and MetaHIT metagenomic datasets for IBD and diabetic patients. Hits (≥24 amino acids and ≥75% ID) were counted and searched against a bacterial genome database to assign taxonomic origins to the hits. Hits assigned to the Firmicute phylum were subdivided based on the species and assigned to 1 of 4 clusters. Values are median abundance of each cluster and error bars are 95% confidence interval. Statistical analysis was Kruskall-wallis (A) or Mann-Whitney U (B). *p<0.05, **p<0.01, ***p<0.001.

**Figure 6:**
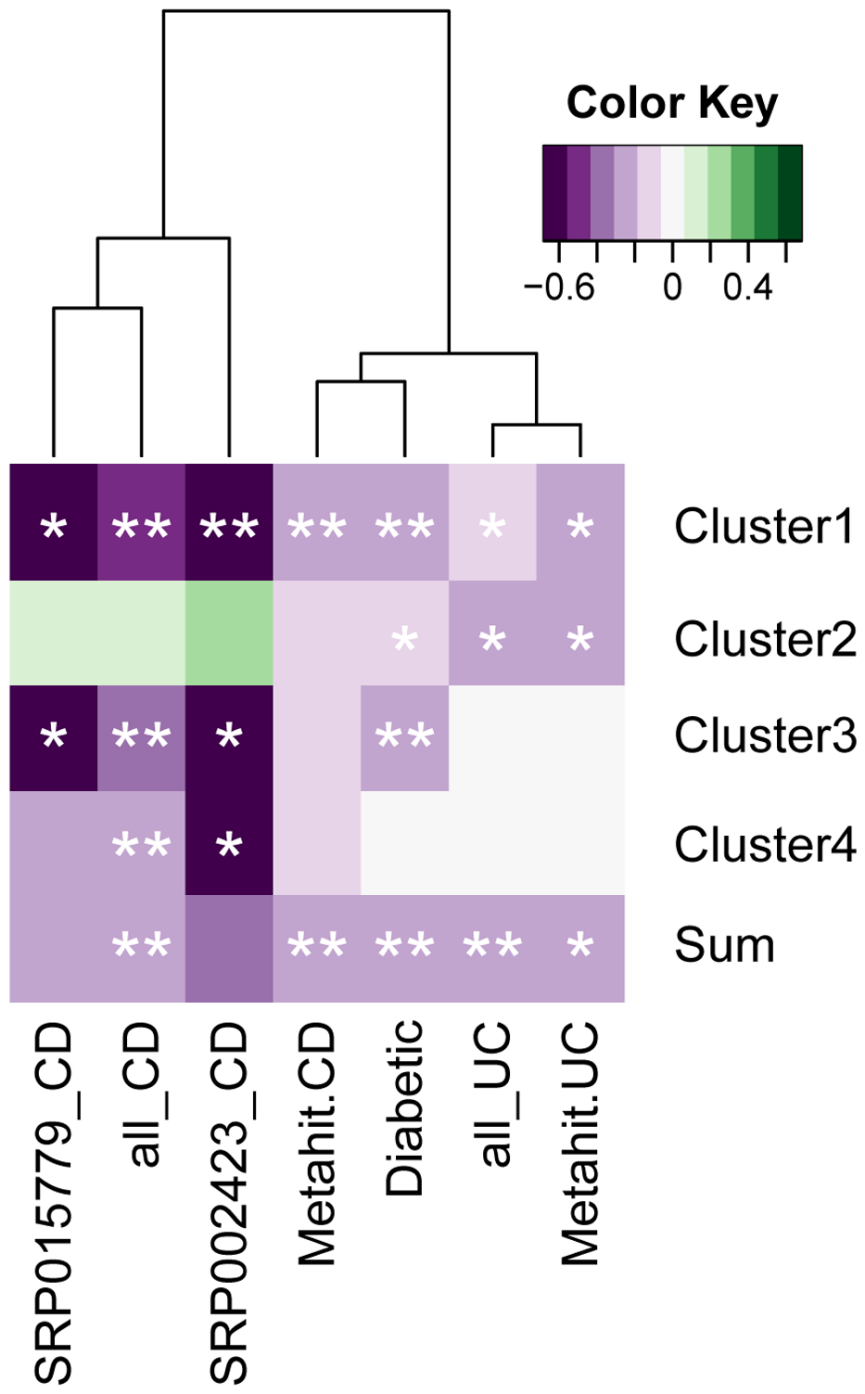
Correlation between the abundance of *bsh* gene assigned to protein clusters and disease. Spearman's rank order correlations was performed and represented using a heatmap to determine the association of *bsh* genes assigned to 4 BSH protein clusters with individual datasets and with the combined dataset for CD, UC or diabetes. “all_CD” and “all_UC” represent the combined datasets while “SRP015779_CD”, “SRP002423_CD”, “Metahit_CD” and “Metahit_UC” represent the correlation of the clusters within these datasets only. Sum represents the correlations of the total abundance of all 4 clusters and the dataset-associated disease. *p<0.05, **p<0.01.

From this observation we proceeded to establish whether a sub group of the Firmicute-derived *bsh* genes may be more strongly associated with disease and whether we could identify it from the protein sequences. An alignment was performed using the protein sequences from *bsh* genes from Firmicutes for the most abundant species found in our datasets (S4 Table). A phylogenetic tree of BSH proteins was created and we identified 2 main clusters and 2 minor clusters representing important groups of Firmicute strains (S1 Figure). An analysis of our database, quantifying the abundance of the *bsh* genes from each of the organisms classified in the 4 clusters (Fig. 5), shows that cluster 1 is significantly reduced in disease states in UC, CD and diabetes (For IBD: Kruskall-Wallis, normal: *n*  =  114, mean rank (MR) 83.15, UC: *n*  =  25, MR 51.40, CD: *n*  =  21, MR 27.36, normal vs CD: *p*  =  0.000, normal vs UC: *p*  =  0.008, and for diabetes: Mann-Whitney U, normal: *n*  =  100, MR  =  112.4, Diabetic: *n*  =  99, MR  =  87.88, *p*  =  0.002). A Spearman's correlation analysis, including an analysis of each datasets separately, further shows the significant relationship between abundance of *bsh* associated with the clusters and disease states (Fig. 6, *p<0.05, **p<0.01). The family and species included in clusters are listed in S4 Table.

## Discussion

To assess the impact of differences in bile metabolism in disease states, we proceeded to analyze the abundance of BA modification genes in metagenomic information from UC, CD and diabetic patients. We found a number of differences between control and diseased populations in the total abundance of genes, the abundance of taxonomically identified genes and in defined phylum-derived genes. We selected a number of datasets for our work as many were of very limited size. One of the largest sets, from the MetaHIT consortium, still included only 4 CD patients. We combined the data using an analysis method normalized based on frequency of BLAST hits against a gene specific database and reported as hits per million sequences analysed. The information obtained can be compared across multiple data sets regardless of the sequencing platform utilized. We further searched for potential control populations in the Human Microbiome Project to include in the analysis. One dataset, of a lean-obese twins study, was included as it provided data from otherwise healthy individuals, mostly from North America.

A previous analysis of the *bsh* gene abundance in the MetaHIT dataset in IBD showed a significant reduction of *bsh* genes derived from Firmicutes in CD patients [26]. This study was however performed on a population of only 4 CD patients. We did not see the same trend in our study, especially when we included patient data from the other metagenomic dataset of IBD and normal patients. We did see significant reduction of *bsh* genes derived from Firmicutes, as a whole, in UC patients but not in CD patients. When we performed statistical analysis on the abundance of *bsh* genes from bacterial families, we noticed that separation strictly at the phylum level did not reflect the complexity of the variability in the sources of *bsh* genes in the population. *Bsh* gene abundance from some bacterial families was positively correlated with disease while others were negatively correlated. By creating subgroups of Firmicute derived *bsh* genes, using a clustering approach of the protein sequences, we identified clusters of strains which showed significant reduction in CD, in UC and in diabetes. Cluster 1, negatively correlated with disease state, includes the *bsh* gene from *Faecalibacterium prausnitzii*, a relatively abundant species associated with health [27]. *F. Prausnitzii bsh* represents however less than 1% of all the *bsh* genes found in cluster 1 in our analysis of the MetaHIT data, the main sources of *bsh* being from *Roseburia intestinalis* (36%), *Erysipelotrichaceae bacterium* (8%) and *Butyrivibrio crossotus* (6%). Interestingly, cluster 1 is largely composed of butyrate-producing bacteria, also positively associated with health [14], [28]. There are multiple lines of evidence, as shown above, that point to BA signals in the pathophysiology of IBD [10]-[12], [18], [29], [30]. IBD is genetically linked, with CD being the most strongly linked, presenting concordance in homozygotic twins of 63.6% [31]. Genetic studies in IBD patients further showed that FXR alleles with reduced signalling or binding to BAs are associated with IBD [11]. FXR involvement in IBD may be related to its regulation of genes dampening intestinal inflammation and bacterial overgrowth [32], [33]. Microbiome analysis changes were also observed in the bacterial population in UC and CD. Decreases in Firmicutes and an increase in Gammaproteobacteria is observed in both UC and CD patients, furthermore, disease is associated with decreased biodiversity [34]. At the genus level, *Roseburia* and *Faecalibacterium* are decreased, which includes the *Lachnospiraceae* and *Ruminococcaceae* families [14], [15], [34]. These changes, especially in *Lachnospiracea* and *Ruminococcacea* may explain some of the reduction seen in Firmicute derived *bsh*, we cannot therefore dismiss whether the changes of *bsh* are mechanistically important or simply associated with other metabolic activity in these organisms.

Our analysis of BSH protein clusters hints to the possibility that BSH activity and specificity may vary between strains, resulting in differential effects on the host. Jones et al. performed an extensive study of the *bsh* genes from the human microbiome. They used a fecal microbiota metagenomic fosmid clone library and identified 142 clones with BSH activity. They identified the phylogenetic divisions of 90 clones and found 30% were derived from Firmicutes, 14.4% from Bacteroidetes, 8.9% from Actinobacteria and the remaining clones (43.3%) could not be associated with a phylum. They also observed that Firmicute and Actinobacteria clones were the only ones able to degrade all conjugated BAs, while the Bacteroidetes BSH seem limited to tauro conjugated [35]. Our observation that clusters of Firmicute derived BSH proteins are differentially associated with disease may support a further level of complexity in the regulation, specificity and activity of BSH proteins encoded within the Firmcute phylum.

Secondary BA modification enzymes originating from Firmicutes, such as dehydroxylase and dehydrogenase, are significantly increased in CD patients. These enzymes, in conjunction with BSH, are important modulators of BA signalling, having significant effects on the affinity of the receptors, and on the capacity of the BAs to enter cells. Structurally, the alpha face of the BA steroid core has several hydroxy groups in positions 3, 7 and 12. The groups in position 7 can directly affect BA binding by interacting with residue rY366 of the FXR receptor. 7-alpha dehydroxylation and 7-epimerization of the CDCA, leading to LCA or UDCA respectively, have the potential to affect FXR signalling by reducing the BA pool capable of providing strong signals. It is also interesting to note that modification of the intestinal microenvironment, especially pH and oxygen tension, can alter the balance of BA populations through changes to the equilibrium of the conversion between CDCA, 7-oxo chenodeoxycholic acid and UDCA [5]. The high abundance of these genes in CD is however at odds with findings that the enzymatic activity from both BSH and ADH are reduced in IBD [12], [18]. The increases observed in these genes in our studies of CD metagenomes may affect signals provided by BAs. Although no such differences could be identified in UC and diabetes, the generation of secondary BAs may still be an important mechanism of signal regulation. The difference observed in *bsh* gene copies may significantly alter the secondary BAs since deconjugation is required prior to dehydroxylation [6].

In our analysis of a large dataset of Chinese control and diabetic patients, we observed a significant reduction in Firmicute-derived *bsh* genes in diabetic group. In support of the importance of BSH in diabetes, Wewalka et al. found elevated levels of tauro-conjugated BAs in T2D patients. Although no significance could be observed in the increased glyco-conjugated BAs, it is hinting strongly to a reduction of the BSH activity, possibly in strains with an enzyme with substrate preference for tauro conjugates [36]. Recent work by Joyce et al, further supports the concept of substrate specificity within *bsh* gene within bacterial strains, with biologically relevant changes observed in the physiology of the animals, in this case a decrease weight gain and improved lipid profile associated with the delivery of a tauro-specific *bsh* gene[37].

Further evidence of the importance of BAs in diabetes was found in a study using vancomycin and amoxicillin to alter the gut microbiome in obese patients with metabolic syndrome. In that study, vancomycin treatment decreased bile acid deconjugation and dehydroxylation activities by reducing Firmicute populations, which resulted in a significant decrease in peripheral insulin sensitivity. A positive correlation was seen between changes in secondary fecal BAs (DCA and LCA) and changes in peripheral insulin sensitivity [38]. Our analysis revealed reduction of 7-ADH and 7-alpha-HSDH derived from Firmicutes in diabetic patients. The deficiency in BA modification genes on the BA pool observed in diabetic populations may be similar to vancomycin treatment since BSH activity is the first step towards dehydroxylation, and that 7-ADH and 7-HSDH genes abundances are also reduced. Our results, and those presented above, are strong indications that BAs are likely to play an important role in diabetes. Another avenue of research showing the potential of BAs to affect diabetes is the observed increase in serum BAs in gastric bypass patients. The increase in serum BA, up to two folds compared to weight matched controls, is positively correlated with serum concentration of GLP-1 and negatively with 2-hour post-prandial glycemia [13], [39]. Almost immediate improvements in glucose homeostasis are seen in the patients, long before the effects on weight can be observed. The mechanism through which BAs are increased is yet to be determined but may be related to a larger proportion of bile reaching the ileum faster, increasing uptake [40].

In this study, we leveraged the metagenomic information available publicly to assess the importance of bile acid modification enzymes in diseases. We showed that significant differences can be found between health and disease, in UC, CD and diabetes. Recent studies support the concept that BAs, in addition to their digestive roles in fat absorption, are of great relevance to provide signals to regulate the overall metabolism and health of the individual, from glucose control to inflammation. Our findings support the idea that the metabolism of BAs, especially deconjugation, may be important in the regulation of these signals and in the modulation of the BA levels in disease states. Further studies may validate the therapeutic use of bile salt hydrolase supplementation in UC, CD and diabetes and justify screening assays to identify such deficiencies in populations at risk for those diseases.

## Supporting Information

S1 Figure **Phylogenetic tree of BSH proteins found in Firmicute bacterial species.** BSH protein sequences were obtained from NCBI protein database, clustered using Clustalw 2.1 and visualized with FigTree 1.3.1. We identified 2 main clusters (c1 and c2) and 2 minor clusters (c3 and c4) representing important groups of Firmicute strains found in our analysis.
(TIF)

S1 Table **Description of the datasets.** Datasets and related information were obtained from the NCBI SRA database. In some datasets, selected samples were excluded if the sequencing did not meet our minimum of 75 bp length. We also limited the datasets to luminal samples from normal, UC, CD or diabetic patients.
(DOCX)

S2 Table **Median abundance of** ***bsh*** **in metagenomic data from individual datasets.** Abundance of *bsh* genes was obtained by searching a BSH protein database against publicly available SRA sequences from HMP and MetaHIT metagenomic datasets for Crohn's Disease (CD) and Ulcerative Colitis (UC). Hits (≥24 amino acids and ≥75% ID) were counted (Total hits) and further searched against a bacterial genome database to assign taxonomic origins to the hits (Assigned hits). Assigned and total hits were quantified and expressed as hits per millions sequences for each individual patient. Values are median abundance for the three datasets including both normal and diseased patients. Number of patients and mean rank in the statistical analysis is presented for each dataset and for both total and assigned hits. Statistical analysis was Kruskall-wallis for multiple groups or Mann-Whitney U for pairwise comparison as indicated.
(DOCX)

S3 Table **7 alpha-dehydroxylase protein IDs for the sequences used for ADH gene search.** A search of NCBI protein database for 7-alpha-dehydroxylase yielded 3 sequences that were identified as 7-alpha-dehydroxylase. Accession numbers, annotation and size of sequences are presented. A recent BLAST search with the sequences reveals further matches assigned to member of the Bacteroidetes, Firmicutes, Actinobacteria and Proteobacteria phyla.
(DOCX)

S4 Table **Bacterial origin of the BSH protein sequences used in clustering with associated clusters.** BSH protein sequences were obtained from NCBI protein database, clustered using Clustalw 2.1 and visualized with FigTree 1.3.1. We identified 2 main clusters (c1 and c2) and 2 minor clusters (c3 and c4) representing important groups of Firmicute strains found in our analysis. Table presents Firmicute families included in the analysis and their corresponding clusters.
(DOCX)
