# Supplementary figures and images for: Bacterial Bile Metabolising Gene Abundance in Crohn's, Ulcerative Colitis and Type 2 Diabetes Metagenomes
Source: PLoS One. 2014 Dec 17;9(12):e115175. doi: 10.1371/journal.pone.0115175 (PMC4269443; doi:10.1371/journal.pone.0115175)

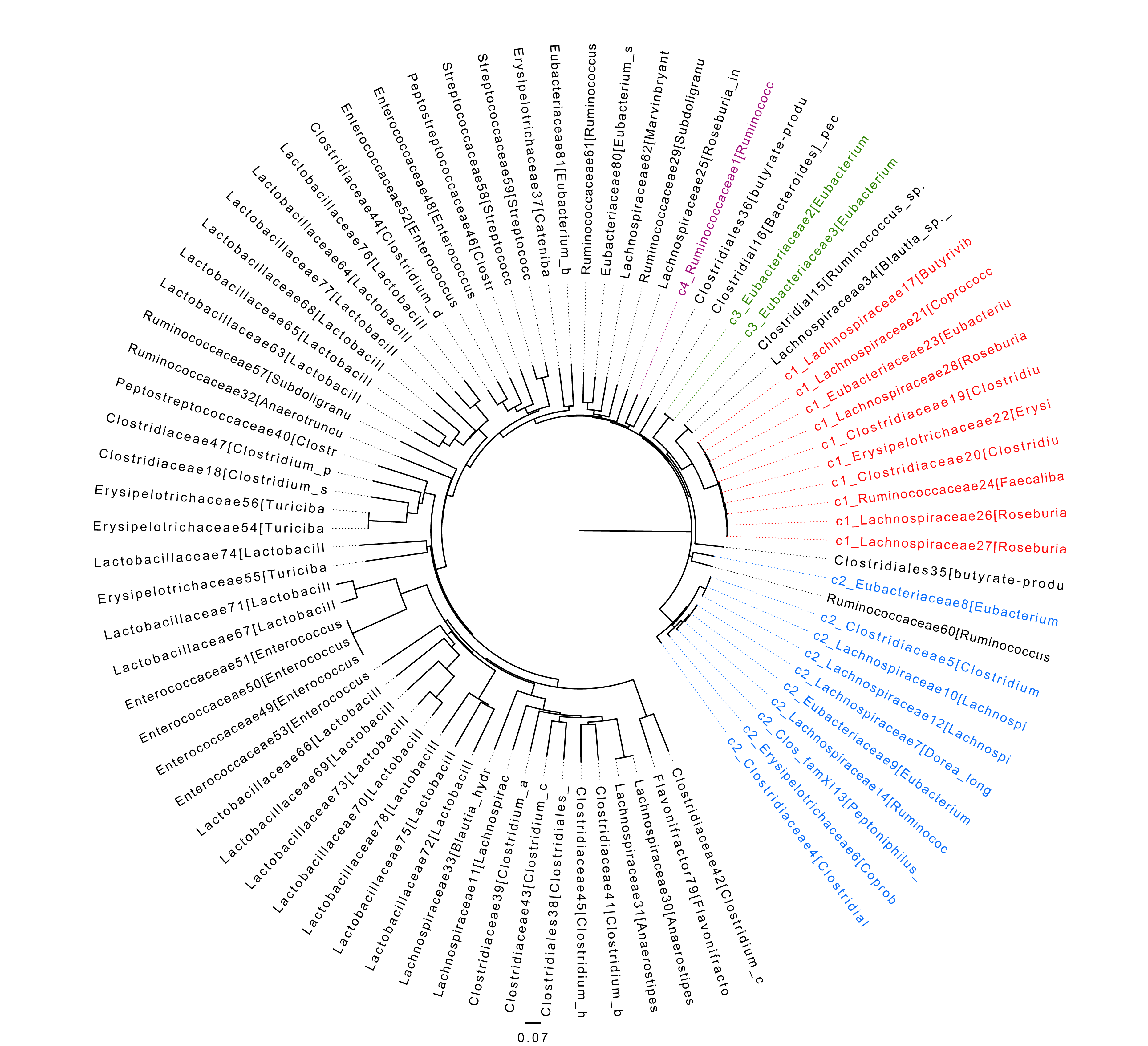

Supplement: S1 Figure — Phylogenetic tree of BSH proteins found in Firmicute bacterial species. BSH protein sequences were obtained from NCBI protein database, clustered using Clustalw 2.1 and visualized with FigTree 1.3.1. We identified 2 main clusters (c1 and c2) and 2 minor clusters (c3 and c4) representing important groups of Firmicute strains found in our analysis. (TIF) [file pone.0115175.s001.tif]
