# Supplementary material for: Bacterial Bile Metabolising Gene Abundance in Crohn's, Ulcerative Colitis and Type 2 Diabetes Metagenomes
Source: PLoS One. 2014 Dec 17;9(12):e115175. doi: 10.1371/journal.pone.0115175 (PMC4269443; doi:10.1371/journal.pone.0115175)
Supplement: S1 Table — Description of the datasets. Datasets and related information were obtained from the NCBI SRA database. In some datasets, selected samples were excluded if the sequencing did not meet our minimum of 75 bp length. We also limited the datasets to luminal samples from normal, UC, CD or diabetic patients. (DOCX) [file pone.0115175.s002.docx]

Table S1. Description of datasets.

| **SRA#** | **Sequencing** | **Sequencing Length*** | **#normal samples** | **#diseased samples** | **Average depth analysed^+^** | **Average insert size** |
| --- | --- | --- | --- | --- | --- | --- |
| **ERP000108** | **Illumina** | **75** | **96** | **21(UC) and 4 (CD)** | **2.3x10^7^** | **130/350** |
| **SRP002427** | **454/illumina** | **~500/125** | **0** | **4 (UC)** | **6x10^5^/1.2x10^7^** | **500/300** |
| **SRP015779** | **Illumina** | **150** | **7** | **4 (CD)** | **5.2x10^5^** | **450** |
| **SRP002423** | **454** | **~500** | **4** | **13 (CD)** | **1.5x10^7^** | **500** |
| **SRP000319** | **454** | **~270** | **18** | **0** | **4.6x10^5^** |  |
| **SRP011011** | **Illumina** | **75/90** | **100** | **99** | **2.3x10^7^** | **350** |

***Exact sequencing length provided for Illumina, approximate for 454.**

**+Average number of sequences for all samples in the datasets, values for SRP002427 are separated by 454 or illumina sequencing.**
