# Supplementary material for: Bacterial Bile Metabolising Gene Abundance in Crohn's, Ulcerative Colitis and Type 2 Diabetes Metagenomes
Source: PLoS One. 2014 Dec 17;9(12):e115175. doi: 10.1371/journal.pone.0115175 (PMC4269443; doi:10.1371/journal.pone.0115175)
Supplement: S2 Table — Median abundance of bsh in metagenomic data from individual datasets. Abundance of bsh genes was obtained by searching a BSH protein database against publicly available SRA sequences from HMP and MetaHIT metagenomic datasets for Crohn's Disease (CD) and Ulcerative Colitis (UC). Hits (≥24 amino acids and ≥75% ID) were counted (Total hits) and further searched against a bacterial genome database to assign taxonomic origins to the hits (Assigned hits). Assigned and total hits were quantified and expressed as hits per millions sequences for each individual patient. Values are median abundance for the three datasets including both normal and diseased patients. Number of patients and mean rank in the statistical analysis is presented for each dataset and for both total and assigned hits. Statistical analysis was Kruskall-wallis for multiple groups or Mann-Whitney U for pairwise comparison as indicated. (DOCX) [file pone.0115175.s003.docx]

Table S2. Median abundance of *bsh* in metagenomic data from individual datasets.

| **Dataset^+^** | **Total or assigned hits** | **Normal**  **median (Mean Rank, n)** | **UC**  **median (Mean Rank, n)** | **CD**  **median (Mean Rank, n)** | **Significance (Mann-Whitney U or Kruskal Wallis)** |
| --- | --- | --- | --- | --- | --- |
| **ERP000108** | **Total** | 216 (56.87, 85) | 213 (50.38, 21) | 205 (53.25, 4) | 0.698 (K-W)* |
|  | **Assigned** | 116 (55.21, 85) | 110 (49.81, 21) | 157 (91.50, 4) | 0.056 (K-W)* |
| **SRP015779** | **Total** | 201 (5.71, 7) | NA | 259 (6.50, 4) | 0.787 (M-W U) |
|  | **Assigned** | 164 (5.00, 7) | NA | 233 (7.75, 4) | 0.230 (M-W U) |
| **SRP002423** | **Total** | 261 (5.75, 4) | NA | 350 (10.00, 13) | 0.163 (M-W U) |
|  | **Assigned** | 210 (5.75, 4) | NA | 283 (10.00, 13) | 0.163 (M-W U) |

***Pairwise comparisons not performed since the K-W test was non-significant.**

**+Other datasets not included as they included too few subjects for statistical analysis.**
