# Supplementary material for: Bacterial Bile Metabolising Gene Abundance in Crohn's, Ulcerative Colitis and Type 2 Diabetes Metagenomes
Source: PLoS One. 2014 Dec 17;9(12):e115175. doi: 10.1371/journal.pone.0115175 (PMC4269443; doi:10.1371/journal.pone.0115175)
Supplement: S3 Table — 7 alpha-dehydroxylase protein IDs for the sequences used for ADH gene search. A search of NCBI protein database for 7-alpha-dehydroxylase yielded 3 sequences that were identified as 7-alpha-dehydroxylase. Accession numbers, annotation and size of sequences are presented. A recent BLAST search with the sequences reveals further matches assigned to member of the Bacteroidetes, Firmicutes, Actinobacteria and Proteobacteria phyla. (DOCX) [file pone.0115175.s004.docx]

Table S3. 7 alpha-dehydroxylase protein IDs for the sequences used for ADH gene search.

| **Seq #** | **Accession** | **Annotation** | **Size** |
| --- | --- | --- | --- |
| **1** | **P07914.3** | **BAIA1_EUBSP7-alpha-dehydroxylase-1/3** | **249** |
| **2** | **P19337.1** | **BAIA2_EUBSP7-alpha-dehydroxylase-2** | **249** |
| **3** | **AAB61153.1** | **7-dehydroxylase_partial-[Clostridium_scindens]** | **55** |
