# Supplementary material for: Bacterial Bile Metabolising Gene Abundance in Crohn's, Ulcerative Colitis and Type 2 Diabetes Metagenomes
Source: PLoS One. 2014 Dec 17;9(12):e115175. doi: 10.1371/journal.pone.0115175 (PMC4269443; doi:10.1371/journal.pone.0115175)
Supplement: S4 Table — Bacterial origin of the BSH protein sequences used in clustering with associated clusters. BSH protein sequences were obtained from NCBI protein database, clustered using Clustalw 2.1 and visualized with FigTree 1.3.1. We identified 2 main clusters (c1 and c2) and 2 minor clusters (c3 and c4) representing important groups of Firmicute strains found in our analysis. Table presents Firmicute families included in the analysis and their corresponding clusters. (DOCX) [file pone.0115175.s005.docx]

Table S4. Bacterial origin of the BSH protein sequences used in clustering with associated clusters.

| Cluster | Bacterial Family* | Specie |
| --- | --- | --- |
| 1 | Lachnospiraceae (17) | Butyrivibrio_crossotus_DSM_2876 |
| 1 | Clostridiaceae (19) | Clostridium_sp._7_3_54FAA |
| 1 | Clostridiaceae (20) | Clostridium_sp._M62/1 |
| 1 | Lachnospiraceae (21) | Coprococcus_comes_ATCC_27758 |
| 1 | Erysipelotrichaceae (22) | Erysipelotrichaceae_bacterium_5_2_54FAA |
| 1 | Eubacteriaceae (23) | Eubacterium_ventriosum_ATCC_27560 |
| 1 | Ruminococcaceae (24) | Faecalibacterium_prausnitzii_A2-165 |
| 1 | Lachnospiraceae (26) | Roseburia_intestinalis_M50/1 |
| 1 | Lachnospiraceae (27) | Roseburia_intestinalis_XB6B4 |
| 1 | Lachnospiraceae (28) | Roseburia_inulinivorans_DSM_16841 |
| 2 | Clostridiaceae (4) | Clostridiales_bacterium_OBRC5-5 |
| 2 | Clostridiaceae (5) | Clostridium_nexile_DSM_1787 |
| 2 | Erysipelotrichaceae (6) | Coprobacillus_sp._8_2_54BFAA |
| 2 | Lachnospiraceae (7) | Dorea_longicatena_DSM_13814 |
| 2 | Eubacteriaceae (8) | Eubacterium_hallii_DSM_3353 |
| 2 | Lachnospiraceae (10) | Lachnospiraceae_bacterium_2_1_58FAA |
| 2 | Lachnospiraceae (12) | Lachnospiraceae_bacterium_3_1_46FAA |
| 2 | Clostridial family XI (13) | Peptoniphilus_sp._oral_taxon_836 |
| 2 | Lachnospiraceae (14) | Ruminococcus_obeum_ATCC_29174 |
| 3 | Eubacteriaceae (2) | Eubacterium_rectale_DSM_17629 |
| 3 | Eubacteriaceae (3) | Eubacterium_rectale_M104/1 |
| 4 | Ruminococcaceae (1) | Ruminococcus_bromii_L2-63 |
|  | Lachnospiraceae (30) | Anaerostipes_caccae |
|  | Lachnospiraceae (31) | Anaerostipes_caccae_DSM_14662 |
|  | Ruminococcaceae (32) | Anaerotruncus_sp._G3(2012) |
|  | Lachnospiraceae (33) | Blautia_hydrogenotrophica_DSM_10507 |
|  | Lachnospiraceae (34) | Blautia_sp._KLE_1732 |
|  | Clostridiales (35) | butyrate-producing_bacterium_SS3/4 |
|  | Clostridiales (36) | butyrate-producing_bacterium_SSC/2 |
|  | Erysipelotrichaceae (37) | Catenibacterium_mitsuokai_DSM_15897 |
|  | Clostridiales (38) | Clostridiales_bacterium_1_7_47FAA |
|  | Clostridiaceae (39) | Clostridium_asparagiforme_DSM_15981 |
|  | Peptostreptococcaceae (40) | Clostridium_bartlettii_DSM_16795 |
|  | Clostridiaceae (41) | Clostridium_bolteae_ATCC_BAA-613 |
|  | Clostridiaceae (42) | Clostridium_cf._saccharolyticum_K10 |
|  | Clostridiaceae (43) | Clostridium_citroniae_WAL-17108 |
|  | Clostridiaceae (44) | Clostridium_difficile_Y384 |
|  | Clostridiaceae (45) | Clostridium_hathewayi_DSM_13479 |
|  | Peptostreptococcaceae (46) | Clostridiumhiranonis |
|  | Clostridiaceae (47) | Clostridium_perfringens |
|  | Enterococcaceae (48) | Enterococcus_casseliflavus_EC30 |
|  | Enterococcaceae (49) | Enterococcus_faecalis_599 |
|  | Enterococcaceae (50) | Enterococcus_faecalis_ATCC_29200 |
|  | Enterococcaceae (51) | Enterococcus_faecalis_EnGen0297 |
|  | Enterococcaceae (52) | Enterococcus_faecium_504 |
|  | Enterococcaceae (53) | Enterococcus_saccharolyticus |
|  | Erysipelotrichaceae (54) | Turicibacter_sanguinis_PC909 |
|  | Erysipelotrichaceae (55) | Turicibacter_sp._HGF1 |
|  | Erysipelotrichaceae (56) | Turicibacter_sanguinis\|WP_006783877.1\| |
|  | Ruminococcaceae (57) | Subdoligranulum_variabile_DSM_15176 |
|  | Streptococcaceae (58) | Streptococcus_infantarius_ATCC_BAA-102 |
|  | Streptococcaceae (59) | Streptococcus_equinusATCC9812 |
|  | Ruminococcaceae (60) | Ruminococcus_lactaris_ATCC_29176 |
|  | Ruminococcaceae (61) | Ruminococcus_callidus_ATCC_27760 |
|  | Lachnospiraceae (62) | Marvinbryantia_formatexigens_DSM_14469 |
|  | Lactobacillaceae (63) | Lactobacillus_vaginalis_ATCC_49540 |
|  | Lactobacillaceae (64) | Lactobacillus_ultunensis_DSM_16047 |
|  | Lactobacillaceae (65) | Lactobacillus_salivarius |
|  | Lactobacillaceae (66) | Lactobacillus_ruminis |
|  | Lactobacillaceae (67) | Lactobacillus_rhamnosus |
|  | Lactobacillaceae (68) | Lactobacillus_reuteri |
|  | Lactobacillaceae (69) | Lactobacillus_plantarum |
|  | Lactobacillaceae (70) | Lactobacillus_parafarraginis_F0439 |
|  | Lactobacillaceae (71) | Lactobacillus_paracasei |
|  | Lactobacillaceae (72) | Lactobacillus_oris |
|  | Lactobacillaceae (73) | Lactobacillus_kisonensis_F0435 |
|  | Lactobacillaceae (74) | Lactobacillus_johnsonii |
|  | Lactobacillaceae (75) | Lactobacillus_hilgardii |
|  | Lactobacillaceae (76) | Lactobacillus_helveticus |
|  | Lactobacillaceae (77) | Lactobacillus_gasseri |
|  | Lactobacillaceae (78) | Lactobacillus_fermentum |
|  | Flavonifractor (79) | Flavonifractor_plautii |
|  | Eubacteriaceae (80) | Eubacterium_siraeum |
|  | Eubacteriaceae (81) | Eubacterium_biforme_DSM_3989 |

* Number in brackets represents the ID used in figure S1.
